# Cases Illustrating the Use of the Elastic Ligature

**Published:** 1874-10-01

**Authors:** 


### CASES ILLUSTRATING THE USE OF THE ELASTIC LIGATURE, IN THE BIRMINGHAM HOSPITAL FOR WOMEN.

*From the London Lancet, Sept.*, 1874.

THE value of the elastic ligature is well illustrated in the subjoined cases. In the first case it was specially suitable. It is, indeed, in the instances of vascular growths so situated that cutting operations are inadmissible, that the gradual strangulation chiefly triumphs. The *modus operandi* tends materially to diminish the risk of hæmorrhage, while in operations of no great magnitude the danger of the supervention of phlebitis is but small, at least not greater than after the use of the knife, the cautery, injections, or the ordinary ligatures.

M. B.——, aged twenty-five, admitted Dec. 25th, with a large varicocele of left labium, which gave rise to much discomfort from the excoriations and discharge. Its removal had been attempted in another institution, but the hæmorrhage had been so alarming that it was not persisted in. According to her own account, the patient had to be watched night and day by dressers.

Feb. 2d.—Mr. Tait passed a double elastic ligature through the base of the tumor by means of a trocar, and tied it in opposite directions, so that each half of the ligature embraced half of the base of the tumor. A quarter of a grain of morphia was at the same time injected under the skin of the arm.

4th.—The tumor quite black, and nearly separated.

7th.—The separation is complete, and a healthy granulating surface about three inches in diameter is left, to which red lotion was applied as a dressing. Very little pain was complained of after the first twenty-four hours, and there was never the least hæmorrhage. On the 23d of the month the wound was almost healed.

H. S——, aged thirty-three, admitted Jan. 5th with a deep and sinuous fistula leading from about two inches to the left of the anus, through the ischio-rectal fossa to an aperture in the rectum, about three inches up. An elastic ligature was passed and tied on the 26th, and it came out on the 30th. Considerable pain was felt for a few hours after its insertion. The track healed perfectly, and the patient was discharged cured. In this case the advantage of the ligature over the knife was that it saved all loss of blood, and, as the patient was very anæmic, that was a point of importance.

M. P——, admitted April 27th, suffering from three perineal fistulæ, one opening to the right of the right labium, and the others about an inch and two inches respectively to the right of the anus. The uterus was quite fixed, and the history given indicated the occurrence of a pelvic hæmatocele some months previously, and its subsequent suppuration. These fistulous tracks led up into a cavity behind the uterus, from which a very abundant discharge flowed after its exploration.

May 1st.—Two elastic ligatures were passed, one through the track opening in the labium, and the other through the principal track to the right of the anus into the suppurating cavity, and thence through an opening made into the rectum, and then they were tied through the rectum. They made their way out on May 4th and 5th. A few days later the discharge was coming entirely through the rectum.

19th.—The discharge has very much diminished in quantity, and the patient is now able to sit down comfortably, as she has not done for many months.

INFLUENCE OF ANÆSTHETICS UPON THE SEXUAL IMPRESSIONS OF FEMALES.—A physician called as an expert before a United States tribunal made the following declaration: "A woman under the influence of anæsthesia is more liable to conception than when sexual intercourse has happened by force, and I concur in the opinion of Dr. Beck, expressed in his treatise on medical jurisprudence, that women may conceive during anæsthesia. The relaxation it produces facilitates conception."

This point seems to me established; but I desire to add an observation which I have made in my practice, and one that it deeply concerns physicians to know. It is well known to-day that occasionally under the influence of ether or chloroform an excitation of the sexual organs is produced and a feeling is excited in the mind by this sensation which may make a woman believe that she has been subjected to violence.

The first case of this nature which I witnessed myself occurred during a delivery. The woman, placed under chloroform, experienced sexual sensations so vivid that she accused me of having violated her and called on her husband for protection. But he had been with her all the time as well as a dozen women who had never quitted the chamber. In a second case I was administering chloroform to a woman to have a tooth extracted, but the physiognomy of the patient showed an expression of venereal excitement so pronounced that I hastened to call in her parents. On awakening she seemed astonished to see herself surrounded by her family, and clearly exhibitited what her impressions had been.

On another occasion a lady of a certain age entered my office in a state of high excitement, and related that she had gone to her surgeon to have a trivial operation performed, to relieve the pain of which she had taken chloroform, and the surgeon had abused her while under its influence. I was persuaded that she had deceived herself, and on examining all the circumstances clearly proved to her that she had been subject to a delusion.

The moral is that physicians should never administer ether or chloroform except in the presence of witnesses. —*Revue Medicale*, Aug. 17, '74.

TETANUS FOLLOWING ABORTION (*The Medical Press and Circular*, June 10, 1874).—Mr. M. A. Boyd reports the case of a primipara, thin, anæmic, and nervous, in whom abortion was produced during the third month, by a fall. The hemorrhage was easily controlled, and the remains of the ovum came away entirely on the third day. She did well until the morning of the sixth day, when trismus made its appearance, together with the characteristic *risus sardonicus;* opisthotonos followed on the next morning, with frequent and painful spasms, and her condition steadily grew worse until her death, which occurred six days after the commencement of the tetanic symptoms. She was treated with large doses of chloral,—nearly an ounce every twenty-four hours,—and with nutrients and stimulants. The tetanus was probably due to irritation of the brain, from deprivation of blood in an already anæmic subject.—*Phil. Med. Times.*

DR. STUART ELDRIDGE, an American physician connected with the medical staff of five large native hospitals on the island of Yesso, Japan, has a large number of native students attending his clinical lectures. He publishes in the Japanese language a bi-monthly illustrated medical journal which finds numerous readers.

---

MEDICAL BOOKS.—Messrs. Jansen, McClurg & Co., 117 & 119 State st., have on hand a large and complete assortment of Medical Books. All new works and new editions received as soon as issued.

---

DAVIS'S CLINICAL LECTURES.—Messrs. H. C. Lea, Philadelphia, have in press a revised and enlarged edition of this work, which is announced for early issue.

